# Detection of novel mutations that cause autosomal dominant retinitis pigmentosa in candidate genes by long-range PCR amplification and next-generation sequencing

**Published:** 2013-03-21

**Authors:** Miguel de Sousa Dias, Imma Hernan, Beatriz Pascual, Emma Borràs, Begoña Mañé, Maria José Gamundi, Miguel Carballo

**Affiliations:** Molecular Genetics Unit, Hospital of Terrassa, Barcelona, Spain

## Abstract

**Purpose:**

To devise an effective method for detecting mutations in 12 genes (*CA4, CRX, IMPDH1, NR2E3, RP9, PRPF3, PRPF8, PRPF31, PRPH2, RHO, RP1*, and *TOPORS*) commonly associated with autosomal dominant retinitis pigmentosa (adRP) that account for more than 95% of known mutations.

**Methods:**

We used long-range PCR (LR-PCR) amplification and next-generation sequencing (NGS) performed in a GS Junior 454 benchtop sequencing platform. Twenty LR-PCR fragments, between 3,000 and 10,000 bp, containing all coding exons and flanking regions of the 12 genes, were obtained from DNA samples of patients with adRP. Sequencing libraries were prepared with an enzymatic (Fragmentase technology) method.

**Results:**

Complete coverage of the coding and flanking sequences of the 12 genes assayed was obtained with NGS, with an average sequence depth of 380× (ranging from 128× to 1,077×). Five previous known mutations in the adRP genes were detected with a sequence variation percentage between 35% and 65%. We also performed a parallel sequence analysis of four samples, three of them new patients with index adRP, in which two novel mutations were detected in *RHO* (p.Asn73del) and *PRPF31* (p.Ile109del).

**Conclusions:**

The results demonstrate that genomic LR-PCR amplification together with NGS is an effective method for analyzing individual patient samples for mutations in a monogenic heterogeneous disease such as adRP. This approach proved effective for the parallel analysis of adRP and has been introduced as routine. Additionally, this approach could be extended to other heterogeneous genetic diseases.

## Introduction

Retinitis pigmentosa (RP) is the most common form of inherited retinopathy, affecting more than 1.5 million people worldwide. RP constitutes a heterogeneous group of inherited degenerative retinal diseases [1-3] characterized by progressive loss of photoreceptor function resulting in night blindness, reduced peripheral vision, decreased visual acuity, abnormal retinal electrophysiology, and pigmentary retinopathy. RP displays all three modes of Mendelian inheritance—autosomal dominant (adRP), autosomal recessive (arRP), and X-linked (XLRP)—as well as a digenic [4] and mitochondrial inheritance [5,6]. Mutations in nearly 20 genes have been associated with adRP [7,8]. In the last two decades, screening for mutations in candidate genes associated with adRP in individual sample patients has been performed in different populations. Different methods, such as single-strand conformational polymorphism, denaturing gradient gel electrophoresis, or denaturing high performance liquid chromatography followed by direct genomic sequencing and, more recently, mutation arrays, have been used in surveys of mutations in patients with adRP [9]. In some Western populations, almost 50% of the patients with adRP are carriers of a known mutation in a candidate gene. Meanwhile, due to the genetic heterogeneity of RP, patients with novel clinically diagnosed adRP should be analyzed for mutations in at least 12 common candidate genes (*CA4, CRX, IMPDH1, NR2E3, RP9, PRPF3, PRPF8, PRPF31, PRPH2, RHO, RP1,* and *TOPORS*), which contain more than 95% of the known mutations that cause adRP [7-10]. Although the use of arrays containing mutations associated with adRP has facilitated the task of screening for mutations in index patients, only previously reported mutations can be checked [9]. At present, screening for mutations in index patients with adRP is a time-consuming and costly task in genetic laboratories.

In current clinical practice, sequencing of candidate genes involved in a disease in individual patient samples is becoming increasingly important to carry out molecular testing. Introduction of the massive DNA next-generation sequencing (NGS) technology is becoming increasingly necessary in sequencing genes to characterize mutations causing a monogenic disease [8,11,12]. Large NGS platforms have been used for massively parallel DNA sequencing of multiple genes in pooled individuals [13,14]. However, the cost and extremely large capacity of these platforms result in a loss of flexibility for the needs of many clinical genetic laboratories. Recently, a scalable Roche 454 GS Junior benchtop sequencing platform was introduced that is feasible for sequencing a subset of genes in individual samples using the NGS technique at assumable costs. Sharing the same core technology as the GS 20 and the GS FLX, the GS Junior combines single-molecule emulsion PCR with pyrosequencing [15,16].

Medical analysis of candidate genes to characterize the mutation that causes a disease currently requires amplification of the exonic and flanking sequences by PCR as a previous step to individual PCR fragment Sanger sequencing. In heterogeneous diseases like RP, where multiple candidate genes are involved, hundreds of individual exonic sequences should be targeted for PCR reactions and sequencing. This involves designing and manipulating numerous primers and reactions to obtain complete coverage of the sequences of interest. Separate PCRs for each region of interest have to be performed, which is costly and time-consuming when hundreds of PCR are required. Multiplex PCR is an attempt to approach this issue [17]. However, multiplex PCR with numerous primer pairs often results in interprimer interactions or an increase in mispriming events that prevent correct amplification [18]. Alternatively, DNA capture of targeted genomic coding and flanking sequences of several genes by hybridization with custom oligonucleotides followed by sequencing in large NGS platforms has been used in molecular diagnostics [12,19].

We aimed to develop a novel, simple, effective method for detecting DNA genomic variations in several genes associated with adRP, and which could then be incorporated without any special equipment into a molecular testing routine. The challenge, therefore, was to introduce rational methods of molecular analysis to study a few patients in a relatively short time and, thus, meet the clinical demand. This approach circumvents the need for a high concentration of patients and the use of large NGS platforms for cost-effective molecular testing.

We have devised an effective approach using long-range PCR amplification and NGS to analyze all coding exons and flanking splice junctions of the 12 “common” genes (which together account for more than 95% of known mutations) associated with adRP. This approach could be extended to other heterogeneous genetic diseases.

## Methods

### Genomic DNA samples

Informed consent was obtained from all patients before the study, which was conducted in accordance with the Declaration of Helsinki and approved by the internal Clinical Research Ethics Committee (CEIC) of the Hospital of Terrassa, Spain. The experiment was divided into two runs, one run featuring a single library per sequencing plate (PicoTiter Plate or PTP) and another run in which multiple libraries were analyzed in parallel. For the single sample run, we generated a chimeric sample composed of a mix of five long-range PCR (LR-PCR) fragments (amplified from five patients), each containing a previously characterized mutation causing adRP (Table 1), plus 15 LR-PCR fragments from a control individual. For the parallel NGS run, a pool of four complete libraries was generated. Three of these four libraries were built using genomic DNA samples from three index patients with uncharacterized adRP. We consider families with adRP those with at least two affected members in successive generations with or without male-to-male transmission. In families without male-to-male transmission, we generalized to adRP those in whom clinical samples of male and female were similar or had previously been excluded XRP-linked with a linkage analysis of known XRP loci. However, a possible XRP-linked with complete penetrance in women in these families should not be discarded [8].

**Table 1:** adRP point mutations previously identified present in the chimeric sample.

Gene	Chromosome–Position	Primary mutation
CA4	17–58235763	c.700G>A
CRX	19–48342562	c.253–15G>A
PRPF31	19–54627950	c.769_770insA
PRPF8	17–1554116	c.6968_6988delGATCCGCAGAGTAAACCTCCC
PRPH2	6–42672290	c.641C>T

The fourth library included the same chimeric sample used in the single sample run, containing the five previously characterized adRP-causing mutations. These five mutations served as a positive control between the single and parallel NGS experiments. In all cases, DNA isolation from peripheral blood lymphocytes was performed automatically with the MagNA Pure Compact Instrument (Roche, Barcelona, Spain) according to the manufacturer’s protocol.

### LR-PCR Amplification from genomic DNA

Genomic reference sequences were obtained from GenBank of 12 genes associated with adRP: *CA4, CRX, IMPDH1, NR2E3, RP9 (PAP1), PRPF3, PRPF8, PRPF31, PRPH2 (RDS), RHO, RP1,* and *TOPORS*. Pairs of primer sets were designed by the Oligo 7.41 (Molecular Biology Insights, Cascade, CO) program, aiming for amplification of the complete coding exons and flanking splice junctions of each gene. To obtain each library, 20 pairs of primers were used in individual LR-PCR amplifications that render DNA fragments between 3,000 and 10,000 bp containing most of the genomic sequences of the study genes. LR-PCR was performed in 50 μl of reaction using 3.75 units of Long Expand *Taq* Polymerase (Roche, Applied Science Barcelona, Spain) following the manufacturer’s recommendations. The primers and LR-PCR conditions used to amplify each DNA fragment are shown in Appendix 1.

A pilot experiment using the 20 LR-PCR DNA fragments was performed. These DNA fragments were purified and analyzed with agarose gel electrophoresis (Appendix 2), and the concentrations measured in an Epoch Microplate Spectrophotometer combined with the Take3 Multi-Volume Plate (Izasa, Barcelona, Spain). Equimolar amounts of DNA fragments were mixed and then used to prepare the library. Five additional PCR reactions of small exonic sequences that possess a long 3′ intron (exon 1 of *CA4, IMPDH1, PAP1,* and *PRPH2,* and exon 2 of *TOPORS*) were required to avoid as much intronic amplification as possible.

### NEBNext dsDNA fragmentase and NGS library preparation

**Figure 1:**
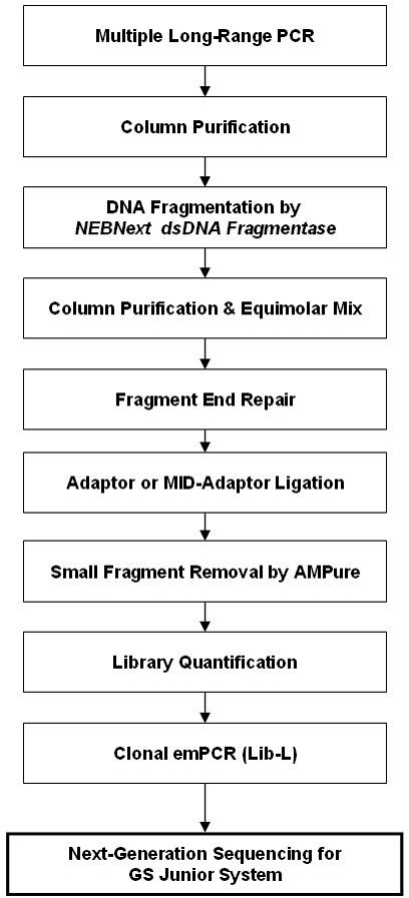
Preparation of LR-PCR fragment library. Workflow representation of a library for NGS prepared from LC-PCR fragments with Fragmentase technology. AMPure® is the trademark of a product used for small fragments removal. Lib-L is a kit for library preparation in NGS.

We assayed an enzymatic method to fragment the DNA of the LR-PCR fragments. The Fragmentase method uses two endonucleases that generate double-stranded deoxyribonucleic acid (dsDNA) breaks that are nearly random and time-dependent. This enzymatic method, outlined in Figure 1, was used to prepare the DNA libraries for sequencing. We preferred to fragment the DNA in a controllable and reproducible enzymatic method. Moreover, no additional instruments or installations are necessary. Nevertheless, before the library was prepared and because of the differences in size and GC content of the 20 LR-PCR fragments, we assayed each fragment in a kinetic reaction with NEBNext Fragmentase enzyme (BioLabs Izasa, Barcelona, Spain). Briefly, the Fragmentase reaction was performed in 50 μl at 0.03 μg/μl of DNA and incubated with 3 μl of enzyme. Aliquots of 10 μl were taken at different times, and the reaction stopped by adding 1 μl of 0.5 M EDTA. Agarose gel electrophoresis of each aliquot was used to analyze the length range of the fragmented DNA. The Fragmentase digestion duration for each DNA fragment that rendered an average length of 750 bp was annotated (Appendix 1).

The 20 LR-PCR fragments obtained for each library were equimolar mixed in three groups according to their reaction time (25, 35, and 45 min). They were then digested with Fragmentase to an average of 750 bp and purified with a High Pure PCR Product Purification kit (Roche Applied Science). The concentration of each fragmented DNA was measured in a microplate spectrophotometer (EPOCH Izasa, Barcelona, Spain). For each library preparation, fragmented DNA was mixed and 120 ng of each mix was end-repaired followed by an adaptor ligation and small fragment removal by AMPure® (Beckman Coulter, Izasa, Barcelona, Spain), as reported in the Rapid Library Preparation Method: GS Junior Titanium Series (Roche) manual.

### Parallel NGS of barcoded samples

To test several samples in parallel in a single sequencing run, four DNA libraries were constructed with LR-PCR followed by Fragmentase digestion, as described above. Each sample library was constructed with a specific adaptor. Each adaptor has a different sequence of ten nucleotides called the molecular identifier (MID) to distinguish each sample after NGS. The DNA library from each sample was pooled, clonally amplified through emulsion PCR (emPCR; GS 454 Junior Method Manual, Roche), and sequenced with the GS Junior platform (Roche).

### Clonal amplification of DNA libraries and NGS

DNA libraries generated by Fragmentase technology need to be clonally amplified for sequencing in the 454 GS Junior platform. Accordingly, single-stranded template and emulsion PCRs were performed according to the emPCR Amplification Method Manual-Lib-L (Roche). DNA sequencing was performed in a GS Junior NGS platform. Preparation of the sequencing run was performed as described in the Sequencing Method Manual (GS Junior Titanium Series; Roche).

We conducted two 454 Junior sequencing runs. In one run, one individual sample of the chimeric library was applied in a single sequencing plate (PicoTiter Plate or PTP). In the other run, equimolar quantities of the tagged fragments for the four samples (chimeric plus three index patients with uncharacterized adRP) were pooled, and a single sequencing reaction was performed. Thus, the four samples were loaded in the same PTP.

### Bioinformatic analysis

We used Roche 454 GS Reference Mapper software (version 2.5p1) to assemble and align the 454 sequencing reads to the gene reference sequences from the GeneBank. After signal processing for Shotgun, reads were mapped to the reference sequence, and “high confidence differences” (HCDiffs) were identified. The criteria for HCDiffs were defined by the GS Reference mapper as variants detected in at least three non-duplicated high-quality reads in forward and reverse reads and found in at least 10% of the total unique sequencing reads (non-duplicate, uniquely mapping reads that align at some location). We also used CLC Genomics Workbench v4.8 (Aarhus, Denmark) as an additional bioinformatics program.

All sequence variants were named according to the Human Genome Variation Society (HGVS) guidelines, using the A of the ATG translation initiation codon as nucleotide + 1. We classified each HCDiff as a single-nucleotide polymorphism or a disease-causing mutation.

## Results

### NGS library construction with LR-PCR and Fragmentase technology

**Figure 2:**
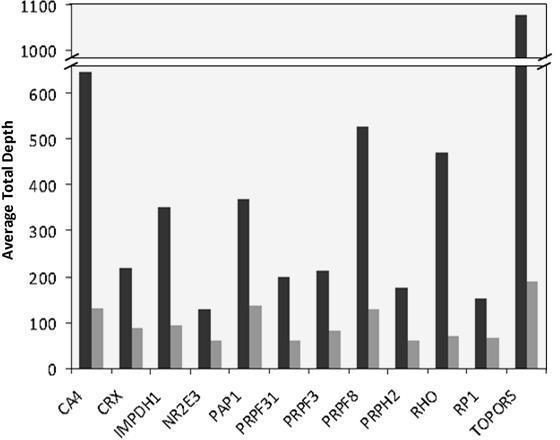
Average total depth for each of the 12 analyzed genes for one sample/PicoPlate (black) and four samples/PicoPlate (gray).

The 12 genes associated with adRP were successfully amplified in 20 fragments (Appendix 1) containing all coding and flanking sequences with LR-PCR. The DNA fragments were used to perform the NGS libraries by Fragmentase technology. Due to the heterogeneous size and sequence (GC content) of the LR-PCR fragments used, a kinetic study for each LR-PCR fragment was performed (Appendix 3). We made an equimolar mix of fragments with similar kinetics and digested them all together in one reaction. Although we showed inter (Figure 2) and intra (Appendix 4) differences in the sequence depth representation of the 12 genes, the coding sequences of the genes were 100% covered with a sequence depth >30×. Thus, the DNA fragmentation by enzymatic method proved effective, as demonstrated by our results. The analysis of the five additional PCR reactions of small exonic sequences with Sanger sequencing showed no sequence variation.

### 454 sequencing analysis

The sequence analysis of the individual GS Junior run of the chimeric library fragmented with Fragmentase was performed using GS Reference Mapper software. A total of 106,000 reads was obtained, of which 72.4% passed filters (% key pass), and 104,000 (98.8%) of these filtered reads were matched with genomic sequences of the 12 genes. Almost 50,000 of these reads (46.7%) were in target coding and flanking sequences. The coverage analysis of the complete coding and 30 nucleotide flanking sequences contained in the LR-PCR fragments showed 100% of the bases covered with an average total depth >30× in the library prepared with Fragmentase (Figure 2). The five previously characterized mutations in *CA4, CRX, PRPF31, PRPF8,* and *PRPH2* presented in the chimeric libraries were also analyzed (Table 2).

**Table 2:** Mutation detection in single and parallel NGS.

Gene	Protein change	Number of samples/Pico titer plate									
		1		4							
		Chimeric sample		Chimeric sample		Sample 1		Sample 2		Sample 3	
		%	Total	%	Total	%	Total	%	Total	%	Total
		Variant	Depth	Variant	Depth	Variant	Depth	Variant	Depth	Variant	Depth
*CA4*	p.Val234Ile	44	280	48	222	0*	-	0*	-	0*	-
*CRX*	None (c.252+11G>A)	50	99	48	67	0*	-	0*	-	0*	-
*PRPF31*	p.Lys257fsX277	47	76	46	57	0*	-	0*	-	0*	-
	p.Ile109del	0*	-	0*	-	47	25	0*	-	0*	-
*PRPF8*	p.Val2325fsX2329	36	101	34	44	0*	-	0*	-	0*	-
*PRPH2*	p.Cys214Tyr	40	48	67	27	0*	-	0*	-	0*	-
	p.Asp338Gly	0*	-	0*	-	100	15	29	17	100	16
	p.Gln304Glu	0*	-	0*	-	96	27	28	25	100	32
	p.Arg310Lys	0*	-	48	40	100	25	92	24	96	28
*RHO*	p.Asn73del	0*	-	0*	-	0*	-	0*	-	38	56
*RP1*	p.Cys2033Tyr	0*	-	0*	-	55	33	50	40	41	83
	p.Asn985Tyr	0*	-	0*	-	60	20	53	17	46	55
	p.Ala1670Thr	0*	-	71	119	0*	-	0*	-	30	54
	p.Ser1691Pro	0*	-	100	122	0*	-	0*	-	49	53

* In the % Variation column the value zero means no variation.

The detection capacity of sequence variants was evaluated by analyzing five previously characterized adRP-causing mutations, carried in the chimeric libraries. These mutations are heterozygous nucleotide substitutions, a deletion of one nucleotide, or small nucleotide deletions, and were detected with a sequence variation from 36% to 50%, with a general sequence depth >48× (Table 2). The sequences also revealed the polymorphism p.Arg310Lys in exon 3 of *PRPH2* [20] in a heterozygous form and two polymorphisms in *RP1,* p.Ala1670Thr and p.Ser1691Pro [21], in a heterozygous and homozygosis form, respectively. These polymorphic variations were validated with Sanger sequencing.

The sequencing analysis of four libraries in parallel was also tested. In this assay, we covered nearly 100% of the bases for almost all twelve study genes (Table 3). However, in the run for sample 2 we detected a lower coverage (49%) of *NR2E3*, suggesting that in the library preparation of this sample the DNA fragment containing the *NR2E3* gene was underestimated. The analysis of sequence depth in the coding and flanking regions of the different genes showed different values (Appendix 4). The differences in sequence depth may suggest unknown sequence effects of Fragmentase or that sequencing specificity could be present. However, graphic representation of the depth and coverage showed a similar gene profile for the one-sample and four-sample sequencing runs, demonstrating the reproducibility of the method for preparing libraries for NGS (Appendix 4).

**Table 3:** Parallel NGS of 12 adRP-associated genes of four samples. Libraries for NGS obtained with Fragmentase technology.

Gene	Chimerical Sample		Sample 1		Sample 2		Sample 3	
	Average	% coverage	Average	% coverage	Average	% coverage	Average	% coverage
	total depth		total depth		total depth		total depth	
	(max-min)		(max-min)		(max-min)		(max-min)	
*CA4*	316	100	50	97	85	100	76	100
	(429–70)		(73–20)		(116–23)		(114–26)	
*CRX*	167	100	42	100	98	100	49	100
	(267–72)		(63–25)		(133–38)		(78–27)	
*IMPDH1*	208	100	43	100	67	100	60	100
	(351–76)		(92–24)		(169–25)		(134–20)	
*NR2E3*	180	100	28	100	21	49	22	100
	(253–54)		(46–21)		(22–20)		(43–20)	
*PAP1*	310	100	81	100	58	100	100	100
	(376–215)		(116–36)		(103–24)		(132–50)	
*PRPF31*	113	100	51	100	32	100	55	100
	(182–42)		(102–25)		(143–27)		(116–21)	
*PRPF3*	140	100	52	100	72	100	68	100
	(216–34)		(187–49)		(203–27)		(156–22)	
*PRPF8*	212	98	73	100	140	96	90	99
	(489–20)		(185–26)		(515–20)		(149–20)	
*PRPH2*	86	100	44	100	52	100	68	100
	(110–39)		(58–29)		(90–24)		(82–38)	
*RHO*	90	100	80	100	38	100	70	100
	(138–41)		(137–49)		(53–21)		(113–36)	
*RP1*	147	100	29	92	23	96	71	96
	(223–28)		(58–20)		(73–20)		(175–20)	
*TOPORS*	213	100	116	100	327	100	110	100
	(281–50)		(187–49)		(507–107)		(179–20)	

In the four-sample parallel run in the GS 454 Junior, the detected variants that could be considered negatives were always in regions containing a poor total sequence depth (less than 20× total depth) or with a total variation below 30%. In the total depth range below 20×, the variant validation as positive or negative may be uncertain because, despite some real heterozygous changes showing values approaching 50% variation, we found some variants with values around 30% that were validated with Sanger sequencing (Table 2). Overall, our results suggest a total depth cut-off sequence of 20×, with 30% to 70% total variation in the heterozygous form and more than 90% in the homozygous form. Accordingly, variants with less than 20× coverage [22] and 30% total variation should not be considered in future sample analyses. However, we showed variants (Table 2) with a depth less than 20× or <30% total variation (i.e., the polymorphisms p.Gln304Glu and p.Asp338Gly in *PRPH2*) that proved to be positive variants after Sanger sequencing validation. A recent overview of factors that contribute to false negative or positive variant calls in the GS-FLX 454 NGS platform [23] suggests considering as potential heterozygous variants those under the cutoff proposed. Thus, following this approach [23], the existing consensus [22] of 20× coverage and 30% used for heterozygous variants would have a power of just 95% [23]. In the context of molecular diagnostics, however, a power of 99.99% should be obtained [23]. To achieve this power, variants with 10× coverage with a 20% variation should be considered. However, the resulting variants with these values must be confirmed with Sanger sequencing.

**Figure 3:**
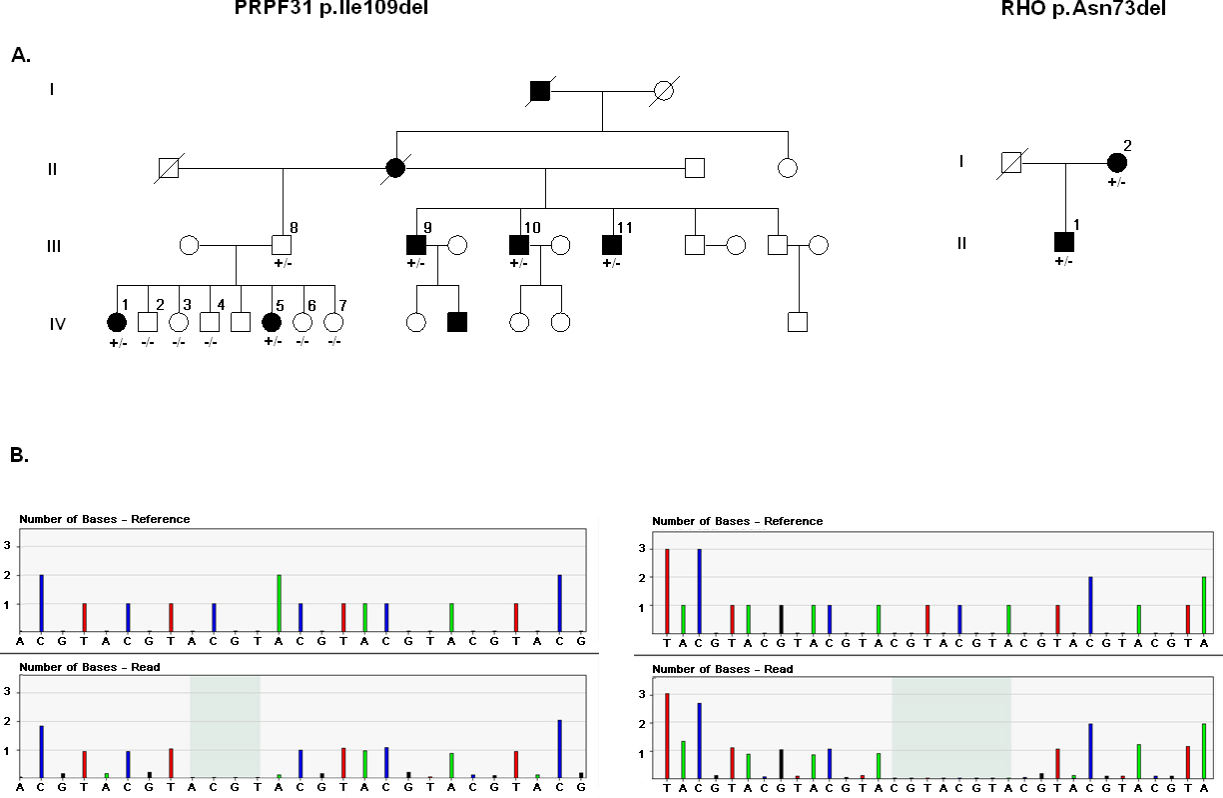
Family segregation of *RHO* (on the right) and *PRPF31* mutations (on the left). **A**: Pedigrees of families carrying mutations in RHO (p.Asn73del) and PRPF31 (p.Ile109del). **B**: Pyrosequencing chromatogram of the mutations p.Asn73del and p.Ile109del. The top plots are idealized flowgrams for the selected reference sequences and the bottom plots are the aligned flowgrams for the selected reads. Each bar represents the signal intensity for each nucleotide and its height corresponds to the number of nucleotides. The deletion sequence is shown when comparing both flowgrams (shadowed region).

Although the average sequence depth obtained decreased compared with the experiment analyzing just one library per PTP, it was enough to detect the control genetic variations present in the chimeric library (Table 2). Additionally, sequencing the other three index patients revealed two novel mutations. In Sample 3, it was possible to detect a deletion of the three nucleotides AAC at position g.312_314 in *RHO*, which causes the novel mutation p.Asn73del in the DNA sample of an index patient (Table 2). In addition, in sequencing Sample 1 we discovered a deletion of the three nucleotides ATC at position g.7092_7094 of *PRPF31*, which causes the novel p.Ile109del mutation. The deletion of the trinucleotide ATC is located in the sixth nucleotide of exon 4 of *PRPF31*, within a putative splicing signal. We checked a possible change in the splicing signal caused by this mutation using two algorithms for splicing prediction (Splice-Site Finder [SSF] Human Splicing Finder version 2.4.1 and Berkeley Drosophila Genome Project (BDGP) NNSPLICE version 0.9). No change in splicing parameters was obtained. Both mutations were confirmed with Sanger sequencing and cosegregated in families with adRP (Figure 3). These mutations were not detected in 215 controls.

In these samples, we also detected four previously reported polymorphisms in *RP1* [21] and three polymorphic variants in exon 3 of *PRPH2* [20] (Table 2). The sequencing highlights the previous finding that one *PRPH2* allele in a Spanish population contains the three polymorphic variations (data not shown). All polymorphic variations were confirmed with Sanger sequencing.

We analyzed the genomic variants found in intronic sequences and found 23 novel intronic variants. These variants were checked with Sanger sequencing and shown to be positive. In addition, these variants were assayed for change in the splicing signal using the two splicing prediction algorithms mentioned above. No changes produced by a single nucleotide or by indels in canonical splicing signals were found (Appendix 5). We also analyzed the intronic sequence data of each gene to search for large homozygous regions. The presence of these homozygous regions could indicate a large deletion in one of the alleles. These deletions, if comprising one or more of the LR-PCR fragments, result as undetectable with our previous gel electrophoresis analysis. The intronic analysis did not show a deletion in the analyzed samples.

## Discussion

Autosomal dominant retinitis pigmentosa is a good example of a monogenic disease with a clear heterogeneity that involves mutations in many genes. We developed a method based on LR-PCR that avoids the use of large numbers of primers and PCR reactions and which results in a significant reduction in cost and time for PCR amplification of all coding and flanking sequences of 12 common genes associated with adRP. Mutations in these genes account for more than 95% of the reported disease-causing mutations associated with adRP. In Western populations, most mutations causing adRP are found in *RHO*, with a percentage of 15%–20% in a Spanish population. In this population (with nearly 200 patients analyzed thus far), the other known adRP genes account for between 0.7% and 5%. Although in previous and recent NGS surveys for adRP we have not detected any mutation in *TOPORS or PAP1* causing RP, we nevertheless included these genes as candidates in our NGS approach. We also included the complete sequence of *NR2E3*, which has previously been associated with recessive RP, because some mutations in this gene have also been reported to be dominant [24-26].

In our design of the LR-PCR fragments, we considered the structure of each gene and the clustering of the coding sequences. Accordingly, we omitted five exons (exon 1 of *CA4, IMPDH1, PAP1,* and *PRPH2* and exon 2 of *TOPORS*) in the LR-PCR fragments because these exons possess a long 3’ intron. It is more cost-effective to analyze these exons for mutations separately with conventional techniques. Alternatively, these exons could be processed as individual amplicons, added to the NGS library pool, and sequenced. The *NRL* gene, a common adRP-associated gene that accounts for 1% of the Spanish adRP population, was not included in this analysis because the genomic structure allows the gene to be analyzed more easily with direct genomic Sanger sequencing.

In our approach, we detected all the control genetic variations that we had previously characterized with conventional screening and Sanger sequencing. We detected the presence of heterozygous variations in a sequence depth >30×. However, as we previously failed to detect some indel mutations with the Reference Mapper software used in the GS Junior platform [27], we also used in parallel CLC v4 software in the bioinformatics sequencing analysis. With this program, we obtained a sequencing profile similar to that seen with Reference Mapper using default settings, except that more variations in homopolymer sequences were found. However, these extra variants proved undetectable when checked with canonical Sanger sequencing.

We first established our cutoff at a minimum total sequence depth of 20× with a sequence variation >30% for a heterozygous variation [22]. However, we detected sequence variants with a total depth below 20× with a sequence variation between 35%–65% that were validated with capillary Sanger sequencing. Thus, in accordance with a recent report [23], a total depth of >10× values should be considered to increase the detection power to near 100%. Our approach detects missense variations and small deletions/insertions (indels), as well as zero false-positive calls in the coding and flanking sequences of any gene in any sample. The 23 novel intronic variants found (Appendix 5) were also validated as real variants with Sanger sequencing. However, our sequence analysis failed for variants in homopolymer stretches larger than six nucleotides. This sequencing limitation has been reported when using pyrosequencing methodologies [28,29]. Nevertheless, we believe that while these analyses are under development, apparently positive results should still be validated with conventional Sanger sequencing [19,27].

Using LR-PCR amplification of genomic DNA, the highest number of sequences obtained corresponds to intronic regions of the genes. These intronic sequences may constitute just background in DNA capturing or be absent in amplicon assay strategies. Nevertheless, in our approach, these intronic sequences comprise the majority (53%) and thus limit the number of samples that can be run in parallel, though this does not compromise the sequencing of coding and flanking sequences. However, some intronic sequence variations may create aberrant splice sites that may generate mutant alleles [30], and annotation of these sequences could be interesting in a future analysis [31]. Our approach for screening for mutations in the common adRP genes demonstrated that using MIDs, at least four samples could be processed in parallel, proving an effective method for analyzing individual novel index patients with adRP, saving time and costs. Moreover, a limited number of novel putative disease-causing variants that are usually obtained must be cosegregated in the family resulting in a limited additional cost in the diagnostic approach. Recently, an effective targeted high-throughput DNA capture and sequencing method has been used to analyze 40 genes associated with RP in isolated cases of RP [12]. However, this approach requires custom arrays and larger platforms for NGS, which would prove more effective in a large survey of patients with unclassified (isolated) RP.

We demonstrated the validity of our method by detection, in index patients with adRP, of two novel mutations that were not detected with commercially available arrays. These genetic variations correspond to two novel mutations that cause adRP. The mutation p.Asn73del in *RHO* was detected in one index patient and his mother, both diagnosed with RP. The Asn-73 residue is conserved among the four proteins, rhodopsin and red, blue, and green opsins [32], suggesting that it plays an important structural or functional role. Moreover, in vitro studies of bovine *Rho* have demonstrated the critical role of Asn-73 in binding with arrestin [32]. The genetic variation detected in *PRPF31* is an ATC deletion at position g.7092_7094, which corresponds to the sixth nucleotide of exon 4. Whether this deletion affects the acceptor signal splicing site of exon 4 remains to be investigated. Cosegregation of this mutation in the available members of the family showed one obligate asymptomatic carrier. However, incomplete penetrance for mutations in *PRPF31* that cause adRP [33] has been reported previously.

The analysis reported here may be extended with new genes associated with adRP that could be processed together with these common genes or, additionally, create a second block of genes associated with adRP that could be analyzed separately using this methodology. Our approach circumvents some limitations found in previous surveys of detection of adRP-causing mutations. Thus, we analyzed for mutations all sequences of known candidate genes. Previously, in most of routine analysis of adRP performed, some regions of these genes in which mutations that cause adRP have never been found, are usually not analyzed. This may cause a bias of mutations in these genes [7-10]. In our approach, we detected not only the previously reported mutations but also the novel mutations that are limited in the array approach. The parallel analysis of four or more patient samples made this approach cost-effective. Moreover, the samples excluded for mutations by this approach comprise a suitable candidate to seek novel genes associated with adRP by a complete NGS exome analysis, for example. Furthermore, we believe that our NGS approach could be used for mutation analysis in other heterogeneous monogenic diseases as well as other diseases where a large number of genes are implicated.

## Appendix 1. Primers, annealing temperature, LR-PCR fragment size and fragmentase digestion times of 12 common genes associated with adRP.

To access the data, click or select the words “Appendix 1.”

## Appendix 2. Electrophoresis of the LR-PCR fragments.

1.0% agarose gel electrophoresis of the long-range PCR fragments obtained using the primers and conditions shown in Appendix 1. Lanes at the left and right ends are molecular DNA markers. To access the data, click or select the words “Appendix 2.”

## Appendix 3. Kinetics of fragmentation of LR-PCR fragments.

Electrophoresis in 1.2% agarose gel of DNA from PRPF31 LR-PCR fragment treated with Fragmentase at different times. Lane MWM is the molecular DNA markers. To access the data, click or select the words “Appendix 3.”

## Appendix 4. Coverage plot of the sequenced LR-PCR fragments.

The plot shows the depth coverage profile of the different fragments-amplified coding and intronic flanking regions (30 bp) for single (dark gray) and multiple (light gray) sample runs. To access the data, click or select the words “Appendix 4.”

## Appendix 5. New mutation detection in the single and parallel NGS for the intronic regions.

In this appendix we listed all 23 novel intronic variation detected by our assay. To access the data, click or select the words “Appendix 5.”
